# Confidence limits for the averted infections ratio estimated via the counterfactual placebo incidence rate

**DOI:** 10.1515/scid-2021-0002

**Published:** 2021-11-24

**Authors:** David T. Dunn, Oliver T. Stirrup, David V. Glidden

**Affiliations:** Institute for Global Health, University College London, London, UK; MRC Clinical Trials Unit, University College London, London, UK; Department of Epidemiology and Biostatistics, University of California San Francisco, San Francisco, CA, USA

**Keywords:** active-control trial, averted infections, confidence limits, non-inferiority

## Abstract

**Objectives:**

The averted infections ratio (AIR) is a novel measure for quantifying the preservation-of-effect in active-control non-inferiority clinical trials with a time-to-event outcome. In the main formulation, the AIR requires an estimate of the counterfactual placebo incidence rate. We describe two approaches for calculating confidence limits for the AIR given a point estimate of this parameter, a closed-form solution based on a Taylor series expansion (delta method) and an iterative method based on the profile-likelihood.

**Methods:**

For each approach, exact coverage probabilities for the lower and upper confidence limits were computed over a grid of values of (1) the true value of the AIR (2) the expected number of counterfactual events (3) the effectiveness of the active-control treatment.

**Results:**

Focussing on the lower confidence limit, which determines whether non-inferiority can be declared, the coverage achieved by the delta method is either less than or greater than the nominal coverage, depending on the true value of the AIR. In contrast, the coverage achieved by the profile-likelihood method is consistently accurate.

**Conclusions:**

The profile-likelihood method is preferred because of better coverage properties, but the simpler delta method is valid when the experimental treatment is no less effective than the control treatment. A complementary Bayesian approach, which can be applied when the counterfactual incidence rate can be represented as a prior distribution, is also outlined.

## Introduction

In a series of papers we have considered the analysis of active-control non-inferiority trials with a time-to-event outcome in the context of HIV prevention trials (Dunn and Glidden 2019; Dunn et al. 2018; Glidden, Stirrup, and Dunn 2020). Our key conclusion is that the standard metric used in such trials, the rate ratio comparing experimental and control arms, is misleading. We further argued that clinically meaningful inference requires estimation or specification of one of two unobserved parameters: (a) the event rate that would have been observed in trial subjects if they had received no treatment (counterfactual placebo arm) or (b) the effectiveness of the control arm relative to the counterfactual placebo arm. With this information, in combination with the observed incidence rates in the control and experimental arms, we can estimate a measure called the averted infections ratio (AIR). The AIR is interpreted as the proportion of events that would be averted by use of the experimental treatment compared with the control treatment. In the context of non-inferiority trials, it is a natural criterion for assessing the degree to which the experimental treatment preserves the effect of the control treatment relative to no treatment (“preservation-of-effect”) (Ghosh et al. 2011; Pigeot et al. 2003; Snapinn and Jiang 2008). Non-inferiority trials using this approach typically aim to demonstrate at least 50% preservation-of-effect, although this value is context specific and higher values may be warranted (Pigeot et al. 2003; Ghosh et al. 2011). In this paper, we consider the derivation of confidence limits for the AIR when it is estimated via the counterfactual placebo incidence.

## Notation and statistical formulation

Denote the hypothetical placebo, control, and experimental arms by the subscripts P, C, and E, respectively. We observe *F*_C_ person-years follow-up in control arm and *F*_E_ person-years follow-up in experimental arm. Let *X*_C_ and *X*_E_ be the random variables denoting the number of observed events, where we assume that $X_{C}∼\text{Poi}\left(F_{C}\lambda_{\text{C}}\right)$ and $X_{\text{E}}∼\text{Poi}\left(F_{E}\lambda_{\text{E}}\right)$. Let *λ*_P_ represent the counterfactual placebo incidence. The averted infections ratio is defined as

(1) $$\Psi =\frac{\lambda_{P}-\lambda_{E}}{\lambda_{P}-\lambda_{C}}$$

Alternatively, Ψ can be expressed in terms of the counterfactual control arm effectiveness ($\left.\theta_{C}=1-\lambda_{C}/\lambda_{P}\right)$ rather than *λ*_P_:

$$\Psi =\frac{1-\lambda_{E}/\lambda_{C}\left(1-\theta_{C}\right)}{\theta_{C}}$$

In this formulation, Ψ is a linear function of the rate ratio and confidence limits for Ψ can be obtained by direct transformation of confidence limits for the rate ratio. As the latter problem has been extensively studied (Graham, Mengersen, and Morton 2003; Li, Tang, and Wong 2014; Price and Bonett 2000; Sahai and Khurshid 1993) we focus on formulation (1).

## Inference conditional on counterfactual incidence

This section considers the derivation of confidence limits for the AIR when considering a single, pre-specified value of *λ*_P_. This allows exploration of how the confidence limits (and point estimates) vary over a range of plausible values of *λ*_P_, which can be highly informative (Glidden, Stirrup, and Dunn 2020).

### Delta method

We first apply a log transformation to the AIR, a natural procedure for any statistic that is a ratio of two variables. From Eq. (1),

$$\log_{e}\;\Psi =\log_{e}\left(\lambda_{P}-\lambda_{E}\right)-\log_{e}\left(\lambda_{P}-\lambda_{C}\right)$$

Based on a first-order Taylor series expansion (Oehlert 1992)

$$var\left(\log_{e}\;\hat{\Psi }\right)\to \frac{var\left({\hat{\lambda }}_{E}\right)}{{\left(\lambda_{P}-{\hat{\lambda }}_{E}\right)}^{2}}+\frac{var\left({\hat{\lambda }}_{C}\right)}{{\left(\lambda_{P}-{\hat{\lambda }}_{C}\right)}^{2}}$$

since *λ*_P_ is regarded as fixed. Thus

(2) $$var\left(\log_{e}\;\hat{\Psi }\right)\to \frac{{\hat{\lambda }}_{E}/F_{E}}{{\left(\lambda_{P}-{\hat{\lambda }}_{E}\right)}^{2}}+\frac{{\hat{\lambda }}_{C}/F_{C}}{{\left(\lambda_{P}-{\hat{\lambda }}_{C}\right)}^{2}}$$

A (1-*α*) confidence interval for Ψ is obtained from

$$\;\exp\left(\log_{e}\;\hat{\Psi }\pm z_{\alpha /2}\sqrt{\frac{{\hat{\lambda }}_{E}/F_{E}}{{\left(\lambda_{P}-{\hat{\lambda }}_{E}\right)}^{2}}+\frac{{\hat{\lambda }}_{C}/F_{C}}{{\left(\lambda_{P}-{\hat{\lambda }}_{C}\right)}^{2}}}\right)$$

### Profile-likelihood method

The log-likelihood under a Poisson model is

(3) $$l\left(\lambda_{C},\lambda_{E}\right)=-F_{C}\lambda_{\text{C}}+X_{\text{C}}\;\log_{e}\left(F_{C}\lambda_{\text{C}}\right)-F_{E}\lambda_{\text{E}}+X_{\text{E}}\;\log_{e}\left(F_{E}\lambda_{E}\right)$$

We can express (3) in terms of Ψ via Eq. (1), noting that a nuisance parameter (either *λ*_C_, or *λ*_E_, or a function of *λ*_C_ and *λ*_E_) is also involved. Denoting this arbitrary nuisance parameter by *ζ*, the profile-likelihood confidence region for Ψ is defined by the set of values (Cole, Chu, and Greenland 2014)

(4) $$\left\{\Psi :2\left[l\left(\hat{\Psi },\hat{\zeta }\right)-l\left(\Psi ,\hat{\zeta }\left(\Psi \right)\right)\right]<\chi_{1-\alpha }^{2}\right\}$$

where

$$l\left(\hat{\Psi },\hat{\zeta }\right)=-X_{\text{C}}+X_{\text{C}}\;\log_{e}\left(X_{\text{C}}\right)-X_{\text{E}}+X_{\text{E}}\;\log_{e}\left(X_{\text{E}}\right)$$

is the unconstrained maximised log-likelihood.

An alternative approach is to parameterise the problem in terms of *λ*_C_ and *λ*_E_ rather than Ψ and *ζ*. We therefore maximise (3) subject to the constraint implied by Eq. (1) for a specified value Ψ*. Re-arranging,

$$g\left(\lambda_{C},\lambda_{E}\right)=\lambda_{E}-\Psi^{*}\lambda_{C}+\lambda_{P}\left(\Psi^{*}-1\right)=0$$

Introducing a Lagrange multiplier ($\left.\beta \right)$, we maximise

(5) $$l\left(\lambda_{C},\lambda_{E}\right)+\beta g\left(\lambda_{C},\lambda_{E}\right)$$

Differentiating (5) with respect to *β*, *λ*_E_, and *λ*_C_ results in a set of three non-linear equations:

$$\lambda_{E}-\Psi^{*}\lambda_{C}+\lambda_{P}\left(\Psi^{*}-1\right)=0,\;-F_{E}\lambda_{E}+X_{E}+\beta \lambda_{E}=0,\;-F_{C}\lambda_{C}+X_{C}-\beta \Psi^{*}\lambda_{C}=0,$$

noting that Ψ* and *λ*_P_ are constants. Using the method of elimination,

$$\lambda_{C}=\frac{y+\sqrt{y^{2}-4\;xz}}{2x},\;\lambda_{E}=\Psi^{*}\lambda_{C}-\lambda_{P}\left(\Psi^{*}-1\right)$$

where

$$x=\Psi^{*}\left(F_{C}+\Psi^{*}F_{E}\right),\;y=\left(\Psi^{*}-1\right)\lambda_{P}\left(F_{C}+\Psi^{*}F_{E}\right)+\Psi^{*}\left(X_{C}+X_{E}\right),\;z=\left(\Psi^{*}-1\right)X_{C}\lambda_{P}$$

The roots of the function implied by (4) were found using the **uniroot** function in R (version 4.02), which utilises the golden-section search procedure combined with parabolic interpolation (code in Appendix).

## Unconditional inference

In addition to exploring how the AIR varies over a range of values of the counterfactual incidence, we may wish to integrate over this parameter to obtain the unconditional distribution of the AIR. Bayesian inference provides a natural framework for this problem. Here we consider the case where trial investigators are able to specify a simple prior distribution for the counterfactual incidence, although more sophisticated approaches which incorporate external information are also possible (Glidden, Stirrup, and Dunn 2020).

Assume that the prior for *λ*_P_ can be specified as a Gamma distribution based on background knowledge. For *λ*_E_ and *λ*_C_, we use weakly informative priors ∼Gamma(0.5,0.001) – this approximates to Jeffrey’s prior (Gelman et al. 1995), and also corresponds to adding 0.5 to the observed number of events as discussed in Section 6. As the Gamma distribution is the conjugate prior for the Poisson model, the posterior distributions for *λ*_E_ and *λ*_C_ are Gamma(*X*_E_ + 0.5, *F*_E_ + 0.001) and Gamma(*X*_C_ + 0.5, *F*_C_ + 0.001), respectively (Gelman et al. 1995). We generate samples from the distributions of *λ*_P_, *λ*_E_, and *λ*_C_, to derive the posterior distribution for the AIR using Eq. (1).

The main application of the AIR is in non-inferiority trials, where it is reasonable to assume that *λ*_C_ < *λ*_P_ since the effectiveness of the control drug will already have been established. Further, the AIR is uninterpretable if *λ*_C_ > *λ*_P_ as this would imply there was no yardstick against which the experimental drug could be compared (nothing to preserve). In most realistic applications it is also reasonable to assume that *λ*_E_ < *λ*_P_ as the experimental drug will have been selected as having some biological activity. It is therefore problematic if the sampled values $\left(\lambda_{P}^{*},\lambda_{C}^{*},\lambda_{E}^{*}\right)$ satisfy either

(6) $$\lambda_{C}^{*}>\lambda_{P}^{*}\;or\;\lambda_{E}^{*}>\lambda_{P}^{*}$$

There are three possible re-sampling strategies: (a) re-sample $\lambda_{P}^{*}$ only (b) re-sample $\left(\lambda_{P}^{*},\lambda_{C}^{*}\right)$ if $\lambda_{C}^{*}>\lambda_{P}^{*}$; re-sample $\left(\lambda_{P}^{*},\lambda_{E}^{*}\right)$ if $\lambda_{E}^{*}>\lambda_{P}^{*}$ (c) re-sample all three values $\left(\lambda_{P}^{*},\lambda_{C}^{*},\lambda_{E}^{*}\right)$. The best strategy is not obvious, and all are explored in the example in Section 7.

## Three arm trials with a placebo arm

Trials are occasionally designed with a placebo arm in addition to the control and experimental arms, thereby providing a direct estimate of *λ*_P_ (Ghosh et al. 2011). The Taylor series approximation (Eq. (2)) requires an additional term to reflect the uncertainty in the estimate of *λ*_P_:

(7) $$var\left(\log_{e}\;\hat{\Psi }\right)\approx \frac{{\hat{\lambda }}_{E}/F_{E}}{{\left({\hat{\lambda }}_{P}-{\hat{\lambda }}_{E}\right)}^{2}}+\frac{{\hat{\lambda }}_{C}/F_{C}}{{\left({\hat{\lambda }}_{P}-{\hat{\lambda }}_{C}\right)}^{2}}+\left({\hat{\lambda }}_{P}/F_{P}\right){\left[\frac{{\hat{\lambda }}_{C}-{\hat{\lambda }}_{E}}{\left({\hat{\lambda }}_{P}-{\hat{\lambda }}_{E}\right)\left({\hat{\lambda }}_{P}-{\hat{\lambda }}_{C}\right)}\right]}^{2}$$

The additional term is generally much smaller than the first two terms and, in expectation, (7) tends towards (2) when *λ*_E_ = *λ*_C_. This leads to a paradoxical finding, namely that the sample size of the placebo group appears to be irrelevant when this equality is assumed (as is commonly the case when designing non-inferiority trials). This paradox is explained by the fact that Ψ = 1 when *λ*_E_ = *λ*_C_ regardless of the value of *λ*_P_. However, the placebo group needs to be large enough in order to ensure that the estimate ${\hat{\lambda }}_{P}$ is sufficiently stable. An interesting, unresolved question is the optimal relative sample size allocation to the three arms. We further note the profile-likelihood approach (Section 3.2) could, in principle, be extended to three arm trials.

## Coverage probabilities

### Methods

Exact coverage probabilities for the lower and upper confidence limits (at nominal coverage probabilities of 1-*α*, for *α* = 0.025, 0.05) were computed using the delta method and profile-likelihood method described in Section 3. For the purposes of exposition we assume *F*_C_ = *F*_E_ = 1, so that *λ*_C_ and *λ*_E_ can be considered as the expected number of events, and *λ*_P_ the expected number of counterfactual events, in each of the two trial arms. The following parameters were examined over a grid of values: Ψ = 0.5(0.1)1.0; *λ*_P_ = 40(20)100; *θ*_C_ = 0.6(0.1)0.9. Exact coverage probabilities were computed by

$$\overset{\infty }{\underset{X_{C}=0}{\sum }}\overset{\infty }{\underset{X_{E}=0}{\sum }}\frac{e^{-\lambda_{C}}{\lambda_{C}}^{X_{C}}}{X_{C}!}\frac{e^{-\lambda_{E}}{\lambda_{E}}^{X_{E}}}{X_{E}!}I\left(X_{C},X_{E}\right)$$

where $\lambda_{C}=\lambda_{P}\left(1-\theta_{C}\right),\;\lambda_{E}=\lambda_{P}\left(1-\psi \theta_{C}\right),\;\;\;and$*I*(*X*_C_, *X*_E_) equals 1 if the lower(upper) confidence limit is less(greater) than Ψ, otherwise equals 0.

The log-likelihood is undefined when either *X*_C_ = 0 or *X*_E_ = 0. However, in contrast with the rate ratio, this is a highly informative outcome in terms of the AIR (even *X*_C_ = 0, *X*_E_ = 0). To avoid this problem, *X*_C_ and *X*_E_ were replaced by *X*_C_ + 0.5 and *X*_E_ + 0.5 before applying the methods of Section 3.2. For consistency, this adjustment was also applied for confidence limits determined by the delta method. The addition of 0.5 resulted in improved coverage estimates under both approaches, as has previously been reported for the rate ratio (Price and Bonett 2000).

## Results

The complete set of coverage probabilities for the lower and upper confidence limits are given in the Appendix. However, the lower confidence limit is of primary interest since this is the comparator for the non-inferiority margin. Also, the upper limit of Ψ may be severely constrained for large values of *θ*_C_. Ψ can be expressed as *θ*_E_/*θ*_C_, so that, for example, Ψ ≤ 1.25 if *θ*_C_ = 0.8, Ψ ≤ 1.11 if *θ*_C_ = 0.9.

**Figure 1:**
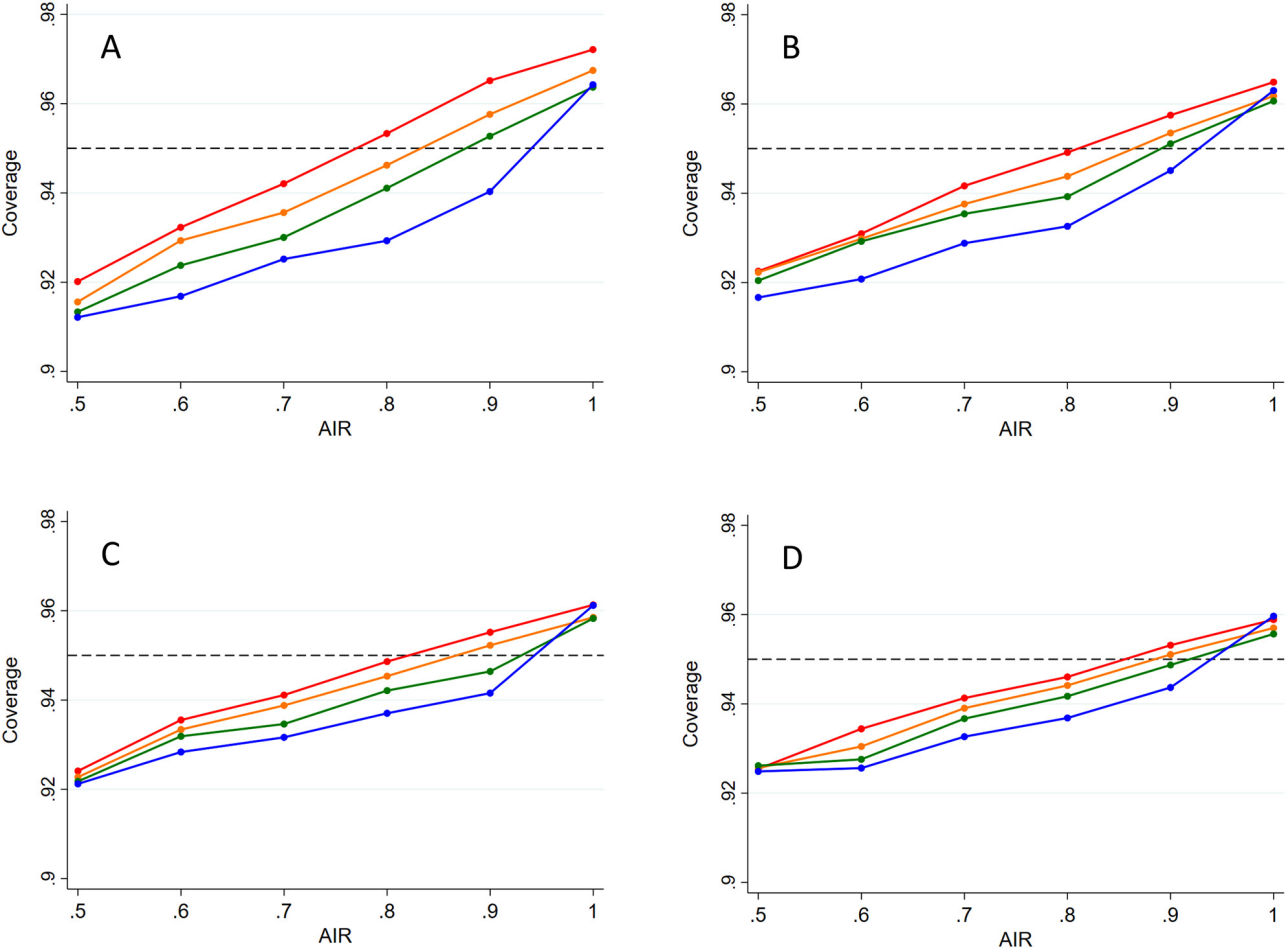
Coverage of lower 5% confidence limit computed using the delta method. (A) 40 expected counterfactual events per arm (B) 60 events (C) 80 events (D) 100 events. Efficacy of control treatment: 0.6, red line; 0.7, orange; 0.8, green; 0.9, blue. Horizontal line represents nominal coverage of 0.95.

Figure 1 shows coverage probabilities using the delta method for the lower one-tailed *α* = 0.05 confidence limit (similar patterns were observed for *α* = 0.025). Coverage is generally too low for Ψ = 0.5–0.8, is reasonably accurate for Ψ = 0.9, and is too high for Ψ = 1.0. This pattern is explained by a negative correlation between the empirical AIR and its estimated standard error, conditional on the true AIR (Ψ). Conditional on Ψ, coverage is higher the larger the value of the control arm effectiveness (*θ*_C_), except for Ψ = 1.0 when differences are minor. As expected, actual and nominal coverage are closer the larger the value of *λ*_P_, although convergence is slow with material discrepancies even for *λ*_P_ = 100. Coverage probabilities for the upper confidence limit were consistently and substantially too high (Appendix), particularly for lower values of Ψ.

Table 1 shows coverage probabilities for the profile-likelihood-based lower confidence limit for *λ*_P_ = 40 and *α* = 0.05. Coverage was close to the nominal value of 0.95 (range 0.9468–0.9615) for all permutations of Ψ and *θ*_C_; as expected, correspondence was even closer at higher values of *λ*_P_ (not shown). Coverage for the profile-likelihood-based upper confidence limit were also highly accurate, in contrast to the delta method (Appendix). The results of these analyses support the routine use of profile-likelihood-based confidence limits, although the delta method is valid in a conservative sense (i.e. actual coverage exceeds nominal coverage) if the true AIR is ≥0.9 approximately. This is reflected in larger values for the lower confidence limit using the delta method (Appendix).

**Table 1:** Coverage probabilities for the profile-likelihood-based lower 5% confidence limit (40 expected counterfactual events per arm).

Effectiveness of control treatment (*θ*_C_)	AIR (Ψ)					
	0.5	0.6	0.7	0.8	0.9	1.0
0.6	0.9468	0.9521	0.9518	0.9522	0.9517	0.9502
0.7	0.9510	0.9539	0.9511	0.9522	0.9519	0.9511
0.8	0.9523	0.9522	0.9553	0.9517	0.9532	0.9518
0.9	0.9539	0.9538	0.9579	0.9489	0.9568	0.9615

Nominal coverage is 0.95.

## Example

The BRIEF TB/A5279 study was a randomised, non-inferiority trial that compared two regimens for the prevention of active tuberculosis in HIV-infected patients who were living in areas of high tuberculosis prevalence or who had evidence of latent tuberculosis infection (Swindells et al. 2019). The reference regimen was 9 months of daily isoniazid alone (9-month arm) and the experimental regimen was 1 month of daily rifapentine plus isoniazid (1-month arm). The incidence of the primary endpoint (diagnosis of tuberculosis, or death from tuberculosis or unknown cause) were similar in the 1-month arm (32 endpoints, 4,926 person-years follow-up (PYFU), incidence rate 0.65 per 100 PYFU) and 9-month arm (33 endpoints, 4,896 PYFU, incidence rate 0.67 per 100 PYFU). The primary metric was the rate difference rather than the rate ratio, which is generally used in HIV prevention research. Non-inferiority was declared by the investigators because the upper 97.5% confidence limit of 0.30 per 100 PYFU was less than the pre-specified margin of 1.25 per 100 PYFU. However, this conclusion is questionable as the authors did not take the counterfactual placebo incidence into account. Notably, the observed incidence in the 9-month arm was markedly lower than the incidence rate assumed for the purposes of sample size calculation (2 per 100 PYFU).

**Figure 2:**
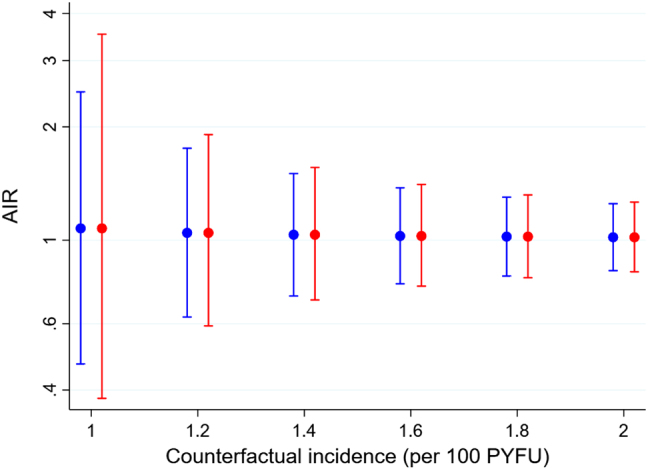
Lower 5% and upper 95% confidence limits for the AIR in the BRIEF TB/A5279 study. Delta method, blue line; profile-likelihood method, red line.

Figure 2 shows the lower 5% and upper 95% confidence limits for the AIR as a function of the counterfactual incidence, computed using the delta and profile-likelihood methods. Consistent with results of Section 6.2, the delta method yields narrower confidence intervals. The figure also reveals the sensitive relationship between the lower confidence limit and the assumed counterfactual incidence, underlining the importance of obtaining as much information as possible about this parameter.

**Figure 3:**
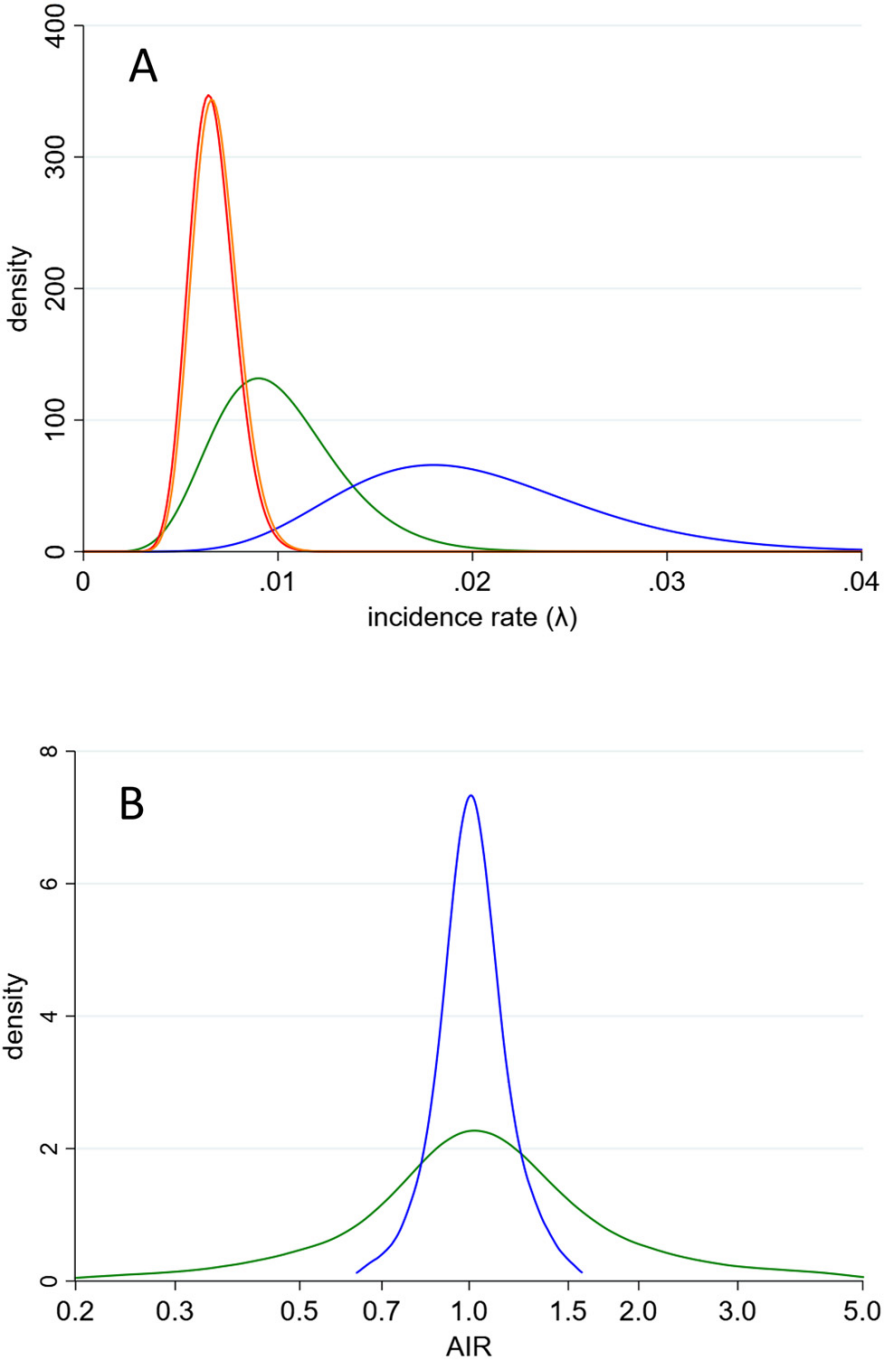
Bayesian analysis of BRIEF TB/A5279 study. (A) Posterior distributions of incidence rate in control regimen (orange line) and experimental regimen (red line); prior distributions of counterfactual incidence (mean = 0.01, green line; mean = 0.02, blue line). (B) Posterior distribution of AIR by prior distribution used for counterfactual incidence (mean = 0.01, green line; mean = 0.02, blue line).

Figure 3 show the results of a Bayesian analysis (10,000 simulations) under two different priors for the counterfactual incidence: Gamma(10,0.001) and Gamma(10,0.002), corresponding to mean incidence rates of 1 and 2 per 100 PYFU, respectively. Without expert knowledge, we emphasise that this is an illustrative rather than a definitive analysis. The lower incidence rate is broadly consistent with the overall ∼30% efficacy of tuberculosis prophylaxis in HIV-infected patients (Ross et al. 2021); the higher value is the rate that the investigators postulated for the control regimen (post hoc, a substantial over-estimate).

For the low incidence scenario, 22.2% of initial simulations had to be re-sampled because of violation of Eq. (6). The posterior median (90% credibility interval) AIR was 1.038 (0.347, 3.627) under re-sampling strategy (a), 1.033 (0.373, 3.228) under strategy (b), and 1.031 (0.357, 3.281) under strategy (c). For the high incidence scenario, only 0.6% of initial simulations had to be re-sampled. The posterior median (90% credibility interval) AIR under re-sampling strategy (a) was 1.009 (0.760, 1.370). The values under the other re-sampling strategies were almost identical (all within ±0.002). In general, our preference is to re-sample $\lambda_{P}^{*}$ only (strategy (a)) since, lacking empirical data, *λ*_P_ is the most uncertain parameter. Figure 3B shows the posterior distributions for the AIR under this strategy, and highlights that inference is much tighter under the high incidence scenario.

## Summary

We have described two approaches for calculating confidence limits for the AIR given a pre-specified value of the counterfactual incidence: a closed-form solution based on a Taylor series expansion (delta method), and an iterative method based on the profile-likelihood, for which R code is provided. The profile-likelihood method is preferred because of better coverage properties, but the delta method is valid when the experimental treatment is no less effective than the control treatment. The difference between the two methods is minimal when the counterfactual incidence is much larger than the observed incidence in both the control and experimental arms. We also describe a simple Bayesian approach when the counterfactual incidence rate can be represented as a simple prior distribution. However, more precise inference can be achieved by harnessing other data which inform the prior distribution (Glidden, Stirrup, and Dunn 2020).

## Supplementary Material

Supplementary Material Details
