# Supplementary material for: Confidence limits for the averted infections ratio estimated via the counterfactual placebo incidence rate
Source: Stat Commun Infect Dis. 2021 Nov 24;13(1):20210002. doi: 10.1515/scid-2021-0002 (PMC9204757; doi:10.1515/scid-2021-0002)

**Supplementary material**

**Confidence limits for the averted infections ratio estimated via the counterfactual placebo incidence rate**

**Dunn DT, Stirrup OT, Glidden DV**

**Content**

1. R code for profile likelihood confidence limits

2. Coverage of confidence limits based on delta method

3. Coverage of upper 5% confidence limit computed using the delta method (Figures)

4. Coverage probabilities for likelihood-based confidence limits with 40 expected counterfactual events per arm

5. Cumulative distribution function of the lower 5% confidence limit (true AIR=1, 40 expected counterfactual events per arm) (Figures)

**1. R code for profile likelihood confidence limits**

# specify the following

X_C<-33+0.5 # addition of 0.5 optional but recommended

X_E<-32+0.5 # ditto

F_C<-4896

F_E<-4926

LAMDA_P<-0.018 # counterfactual incidence

z<-qnorm(0.95) # level of confidence bound

# derived parameters

R_C <- X_C/F_C

R_E <- X_E/F_E

AIR <- (LAMDA_P-R_E)/(LAMDA_P-R_C)

PLL <- function(X,LAMDA_P,X_C,X_E,F_C,F_E,z) { # X is AIR

A<-X*(F_C+X*F_E)

B<-((F_C+X*F_E)*LAMDA_P*(X-1)+X*(X_E+X_C))

C<-X_C*LAMDA_P*(X-1)

R_C<-(B+sqrt(B^2-4*A*C))/(2*A)

R_E<-X*R_C-LAMDA_P*(X-1)

LL_MAX<-(-X_C)+X_C*log(X_C)-X_E+X_E*log(X_E)

LL<-(-F_C)*R_C+X_C*log(F_C*R_C) - F_E*R_E+X_E*log(F_E*R_E)

2*(LL_MAX-LL)-z^2

}

uniroot(PLL,c(0.001,AIR),LAMDA_P=LAMDA_P,X_C=X_C,X_E=X_E,F_C=F_C,F_E=F_E,z=z, tol=1.0e-6)$root # lower bound

uniroot(PLL,c(AIR,100),LAMDA_P=LAMDA_P,X_C=X_C,X_E=X_E,F_C=F_C,F_E=F_E,z=z, tol=1.0e-6)$root # upper bound

####

# following codes plots profile likelihood and Taylor series approximation

PLL_ed <- function(X,LAMDA_P,X_C,X_E,F_C,F_E) { # X is AIR

A<-X*(F_C+X*F_E)

B<-((F_C+X*F_E)*LAMDA_P*(X-1)+X*(X_E+X_C))

C<-X_C*LAMDA_P*(X-1)

R_C<-(B+sqrt(B^2-4*A*C))/(2*A)

R_E<-X*R_C-LAMDA_P*(X-1)

LL_MAX<--X_C+X_C*log(X_C)-X_E+X_E*log(X_E)

LL<--F_C*R_C+X_C*log(F_C*R_C) - F_E*R_E+X_E*log(F_E*R_E)

2*(LL_MAX-LL)

}

Taylor_approx_fun_logscale <- function(X,LAMDA_P,X_C,X_E,F_C,F_E, logscale_SE) { # X is AIR

AIR_max<- (X_E/F_E - LAMDA_P)/(X_C/F_C - LAMDA_P)

2*(log(dnorm(0))-log(dnorm((log(X)-log(AIR_max))/logscale_SE)))

}

logscale_SE<-sqrt( (R_E/F_E)/(LAMDA_P-R_E)^2 + (R_C/F_C)/(LAMDA_P-R_C)^2 )

library("ggplot2")

ggplot() + xlim(0.5, 1.5) + geom_function(fun = PLL, args=list(LAMDA_P=LAMDA_P,X_C=X_C,X_E=X_E,F_C=F_C,F_E=F_E,z=z))

ggplot() + xlim(0.5, 1.5) + geom_function(fun = PLL_ed, args=list(LAMDA_P=LAMDA_P,X_C=X_C,X_E=X_E,F_C=F_C,F_E=F_E)) +

geom_hline(yintercept=z^2, linetype="dashed") + theme_bw() +

xlab("AIR value") + ylab("Profile likelihood difference from max.") +

geom_function(fun = Taylor_approx_fun_logscale, args=list(LAMDA_P=LAMDA_P,X_C=X_C,X_E=X_E,F_C=F_C,F_E=F_E, logscale_SE=logscale_SE), linetype="dotted")

**2. Coverage of confidence limits based on delta method**

Values in the table show actual coverage relative to nominal coverage. For example, for AIR=0.5,θ_C=0.6,λ_P=40, the coverage for the lower 0.025 CL is 0.975-0.0215=0.9535.

+---------------------------------------------------------+

| | | | Lower CL | Upper CL |

| λ_P | θ_C | AIR | 0.025 | 0.05 | 0.95 | 0.975 |

|-----+-----+-----+---------+---------+---------+---------|

| 40 | .6 | .5 | -0.0215 | -0.0299 | +0.0500 | +0.0250 |

| 40 | .6 | .6 | -0.0118 | -0.0177 | +0.0500 | +0.0250 |

| 40 | .6 | .7 | -0.0015 | -0.0079 | +0.0500 | +0.0250 |

| 40 | .6 | .8 | +0.0065 | +0.0033 | +0.0474 | +0.0250 |

| 40 | .6 | .9 | +0.0140 | +0.0152 | +0.0366 | +0.0239 |

| 40 | .6 | 1 | +0.0184 | +0.0221 | +0.0221 | +0.0184 |

|-----+-----+-----+---------+---------+---------+---------|

| 40 | .7 | .5 | -0.0260 | -0.0344 | +0.0500 | +0.0250 |

| 40 | .7 | .6 | -0.0177 | -0.0207 | +0.0500 | +0.0250 |

| 40 | .7 | .7 | -0.0088 | -0.0144 | +0.0495 | +0.0250 |

| 40 | .7 | .8 | -0.0013 | -0.0038 | +0.0421 | +0.0249 |

| 40 | .7 | .9 | +0.0088 | +0.0076 | +0.0303 | +0.0216 |

| 40 | .7 | 1 | +0.0140 | +0.0175 | +0.0175 | +0.0140 |

|-----+-----+-----+---------+---------+---------+---------|

| 40 | .8 | .5 | -0.0284 | -0.0367 | +0.0500 | +0.0250 |

| 40 | .8 | .6 | -0.0226 | -0.0262 | +0.0500 | +0.0250 |

| 40 | .8 | .7 | -0.0157 | -0.0200 | +0.0475 | +0.0250 |

| 40 | .8 | .8 | -0.0069 | -0.0089 | +0.0374 | +0.0243 |

| 40 | .8 | .9 | +0.0038 | +0.0027 | +0.0274 | +0.0195 |

| 40 | .8 | 1 | +0.0130 | +0.0137 | +0.0137 | +0.0130 |

|-----+-----+-----+---------+---------+---------+---------|

| 40 | .9 | .5 | -0.0338 | -0.0379 | +0.0500 | +0.0250 |

| 40 | .9 | .6 | -0.0254 | -0.0331 | +0.0500 | +0.0250 |

| 40 | .9 | .7 | -0.0219 | -0.0248 | +0.0444 | +0.0250 |

| 40 | .9 | .8 | -0.0112 | -0.0207 | +0.0369 | +0.0238 |

| 40 | .9 | .9 | -0.0026 | -0.0097 | +0.0293 | +0.0205 |

| 40 | .9 | 1 | +0.0160 | +0.0142 | +0.0142 | +0.0160 |

|-----+-----+-----+---------+---------+---------+---------|

| 60 | .6 | .5 | -0.0236 | -0.0275 | +0.0500 | +0.0250 |

| 60 | .6 | .6 | -0.0138 | -0.0191 | +0.0500 | +0.0250 |

| 60 | .6 | .7 | -0.0058 | -0.0083 | +0.0482 | +0.0250 |

| 60 | .6 | .8 | +0.0015 | -0.0009 | +0.0374 | +0.0243 |

| 60 | .6 | .9 | +0.0080 | +0.0075 | +0.0250 | +0.0193 |

| 60 | .6 | 1 | +0.0133 | +0.0149 | +0.0149 | +0.0133 |

|-----+-----+-----+---------+---------+---------+---------|

| 60 | .7 | .5 | -0.0259 | -0.0277 | +0.0500 | +0.0250 |

| 60 | .7 | .6 | -0.0164 | -0.0202 | +0.0499 | +0.0250 |

| 60 | .7 | .7 | -0.0105 | -0.0124 | +0.0425 | +0.0250 |

| 60 | .7 | .8 | -0.0029 | -0.0062 | +0.0302 | +0.0222 |

| 60 | .7 | .9 | +0.0038 | +0.0035 | +0.0204 | +0.0161 |

| 60 | .7 | 1 | +0.0107 | +0.0118 | +0.0118 | +0.0107 |

|-----+-----+-----+---------+---------+---------+---------|

| 60 | .8 | .5 | -0.0275 | -0.0296 | +0.0500 | +0.0250 |

| 60 | .8 | .6 | -0.0188 | -0.0208 | +0.0481 | +0.0250 |

| 60 | .8 | .7 | -0.0154 | -0.0146 | +0.0379 | +0.0243 |

| 60 | .8 | .8 | -0.0074 | -0.0108 | +0.0283 | +0.0205 |

| 60 | .8 | .9 | +0.0001 | +0.0011 | +0.0195 | +0.0151 |

| 60 | .8 | 1 | +0.0089 | +0.0107 | +0.0107 | +0.0089 |

|-----+-----+-----+---------+---------+---------+---------|

| 60 | .9 | .5 | -0.0288 | -0.0333 | +0.0500 | +0.0250 |

| 60 | .9 | .6 | -0.0219 | -0.0292 | +0.0439 | +0.0250 |

| 60 | .9 | .7 | -0.0199 | -0.0212 | +0.0378 | +0.0232 |

| 60 | .9 | .8 | -0.0177 | -0.0174 | +0.0282 | +0.0202 |

| 60 | .9 | .9 | -0.0031 | -0.0049 | +0.0242 | +0.0171 |

| 60 | .9 | 1 | +0.0104 | +0.0130 | +0.0130 | +0.0104 |

|-----+-----+-----+---------+---------+---------+---------|

| 80 | .6 | .5 | -0.0211 | -0.0259 | +0.0500 | +0.0250 |

| 80 | .6 | .6 | -0.0129 | -0.0145 | +0.0497 | +0.0250 |

| 80 | .6 | .7 | -0.0062 | -0.0089 | +0.0421 | +0.0249 |

| 80 | .6 | .8 | +0.0001 | -0.0014 | +0.0295 | +0.0219 |

| 80 | .6 | .9 | +0.0052 | +0.0052 | +0.0197 | +0.0161 |

| 80 | .6 | 1 | +0.0098 | +0.0113 | +0.0113 | +0.0098 |

|-----+-----+-----+---------+---------+---------+---------|

| 80 | .7 | .5 | -0.0223 | -0.0273 | +0.0500 | +0.0250 |

| 80 | .7 | .6 | -0.0166 | -0.0166 | +0.0465 | +0.0250 |

| 80 | .7 | .7 | -0.0094 | -0.0112 | +0.0351 | +0.0236 |

| 80 | .7 | .8 | -0.0029 | -0.0046 | +0.0257 | +0.0188 |

| 80 | .7 | .9 | +0.0027 | +0.0023 | +0.0164 | +0.0135 |

| 80 | .7 | 1 | +0.0078 | +0.0085 | +0.0085 | +0.0078 |

|-----+-----+-----+---------+---------+---------+---------|

| 80 | .8 | .5 | -0.0234 | -0.0282 | +0.0500 | +0.0250 |

| 80 | .8 | .6 | -0.0172 | -0.0181 | +0.0403 | +0.0249 |

| 80 | .8 | .7 | -0.0117 | -0.0154 | +0.0323 | +0.0217 |

| 80 | .8 | .8 | -0.0059 | -0.0079 | +0.0221 | +0.0175 |

| 80 | .8 | .9 | -0.0008 | -0.0036 | +0.0167 | +0.0127 |

| 80 | .8 | 1 | +0.0069 | +0.0083 | +0.0083 | +0.0069 |

|-----+-----+-----+---------+---------+---------+---------|

| 80 | .9 | .5 | -0.0251 | -0.0288 | +0.0473 | +0.0250 |

| 80 | .9 | .6 | -0.0219 | -0.0216 | +0.0364 | +0.0240 |

| 80 | .9 | .7 | -0.0176 | -0.0184 | +0.0303 | +0.0213 |

| 80 | .9 | .8 | -0.0118 | -0.0130 | +0.0260 | +0.0179 |

| 80 | .9 | .9 | -0.0061 | -0.0085 | +0.0205 | +0.0144 |

| 80 | .9 | 1 | +0.0071 | +0.0112 | +0.0112 | +0.0071 |

|-----+-----+-----+---------+---------+---------+---------|

| 100 | .6 | .5 | -0.0190 | -0.0246 | +0.0500 | +0.0250 |

| 100 | .6 | .6 | -0.0142 | -0.0156 | +0.0476 | +0.0250 |

| 100 | .6 | .7 | -0.0066 | -0.0087 | +0.0362 | +0.0240 |

| 100 | .6 | .8 | -0.0011 | -0.0040 | +0.0245 | +0.0191 |

| 100 | .6 | .9 | +0.0040 | +0.0031 | +0.0164 | +0.0136 |

| 100 | .6 | 1 | +0.0078 | +0.0089 | +0.0089 | +0.0078 |

|-----+-----+-----+---------+---------+---------+---------|

| 100 | .7 | .5 | -0.0207 | -0.0244 | +0.0499 | +0.0250 |

| 100 | .7 | .6 | -0.0156 | -0.0195 | +0.0399 | +0.0249 |

| 100 | .7 | .7 | -0.0087 | -0.0110 | +0.0302 | +0.0213 |

| 100 | .7 | .8 | -0.0038 | -0.0059 | +0.0226 | +0.0166 |

| 100 | .7 | .9 | +0.0019 | +0.0011 | +0.0144 | +0.0114 |

| 100 | .7 | 1 | +0.0063 | +0.0070 | +0.0070 | +0.0063 |

|-----+-----+-----+---------+---------+---------+---------|

| 100 | .8 | .5 | -0.0214 | -0.0239 | +0.0468 | +0.0250 |

| 100 | .8 | .6 | -0.0163 | -0.0224 | +0.0346 | +0.0236 |

| 100 | .8 | .7 | -0.0114 | -0.0133 | +0.0264 | +0.0196 |

| 100 | .8 | .8 | -0.0063 | -0.0083 | +0.0231 | +0.0154 |

| 100 | .8 | .9 | -0.0008 | -0.0013 | +0.0134 | +0.0113 |

| 100 | .8 | 1 | +0.0053 | +0.0057 | +0.0057 | +0.0053 |

|-----+-----+-----+---------+---------+---------+---------|

| 100 | .9 | .5 | -0.0218 | -0.0251 | +0.0407 | +0.0250 |

| 100 | .9 | .6 | -0.0172 | -0.0244 | +0.0319 | +0.0220 |

| 100 | .9 | .7 | -0.0143 | -0.0173 | +0.0264 | +0.0188 |

| 100 | .9 | .8 | -0.0104 | -0.0132 | +0.0202 | +0.0158 |

| 100 | .9 | .9 | -0.0037 | -0.0063 | +0.0174 | +0.0124 |

| 100 | .9 | 1 | +0.0056 | +0.0096 | +0.0096 | +0.0056 |

+---------------------------------------------------------+

**3. Coverage of upper 5% confidence limit computed using the delta method**

40 expected counterfactual events per arm
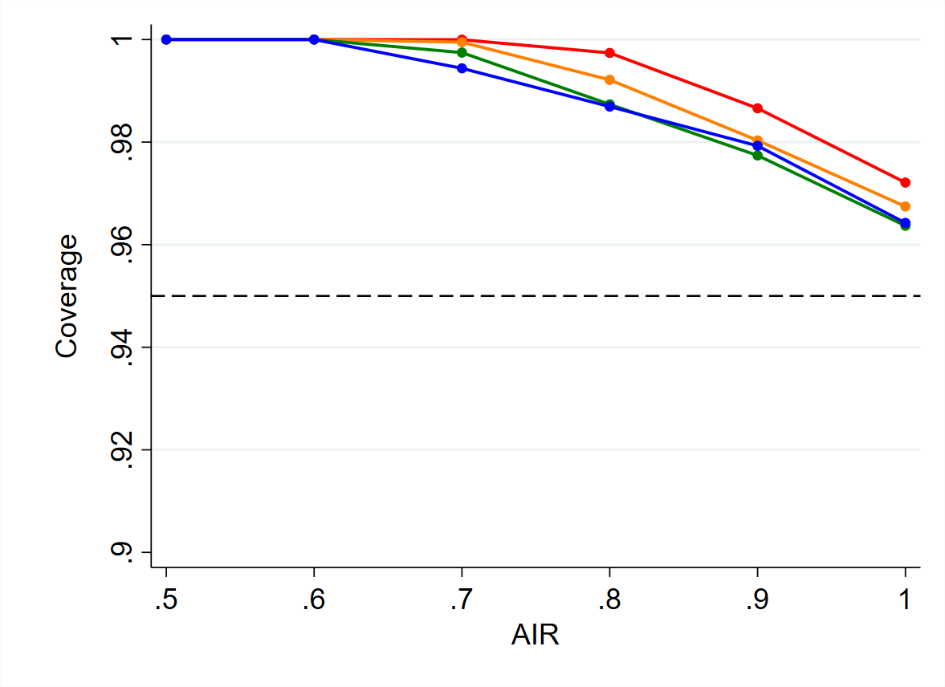


60 expected counterfactual events per arm


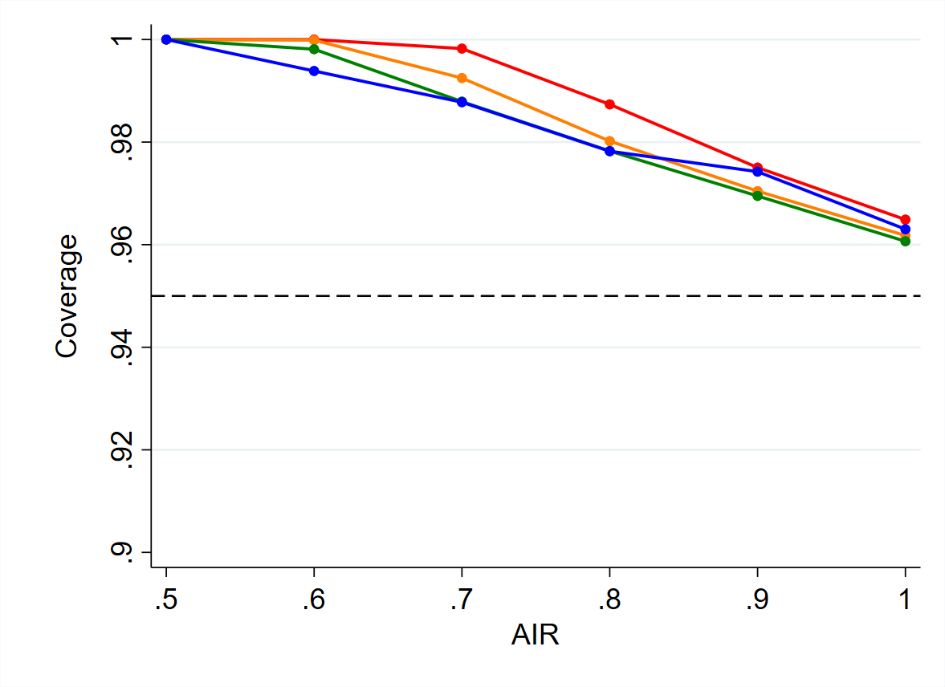


80 expected counterfactual events per arm


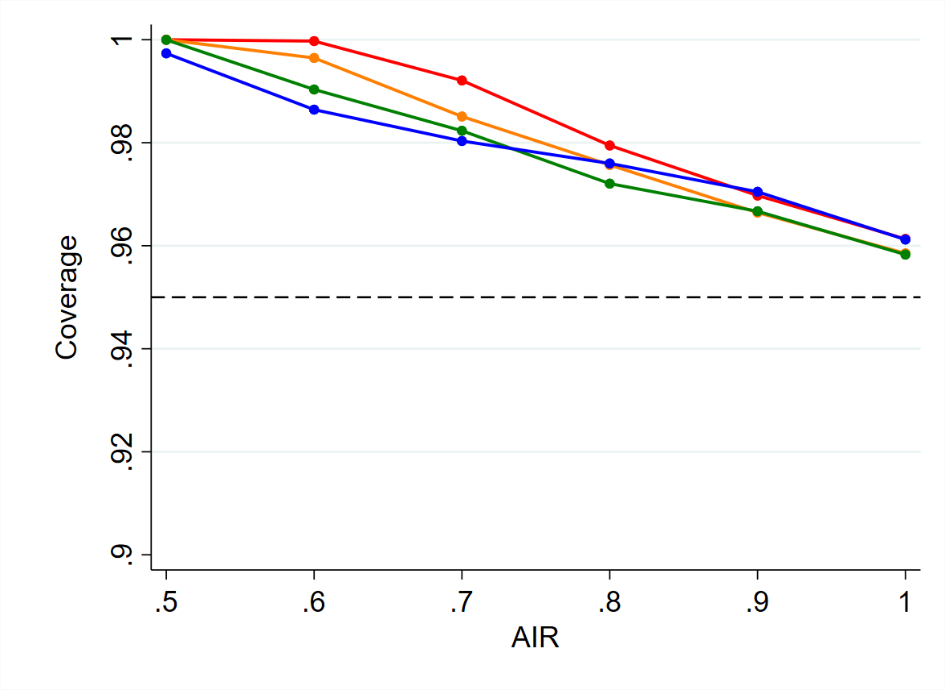


100 expected counterfactual events per arm
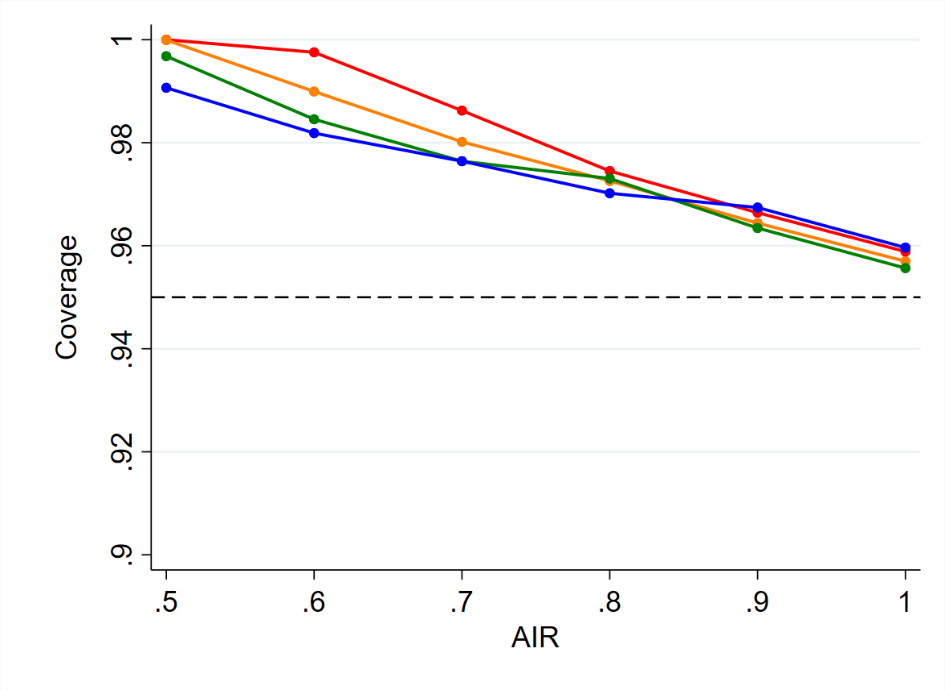


**4. Coverage probabilities for likelihood-based confidence limits with 40 expected counterfactual events per arm**

Upper 95% confidence limit (nominal coverage 0.95)

| Effectiveness of control regimen(Θ_C_) | AIR (Ψ) | | | | | |
| --- | --- | --- | --- | --- | --- | --- |
|  | 0.5 | 0.6 | 0.7 | 0.8 | 0.9 | 1.0 |
| 0.6 | 0.9661 | 0.9539 | 0.9502 | 0.9459 | 0.9521 | 0.9502 |
| 0.7 | 0.9562 | 0.9503 | 0.9493 | 0.9484 | 0.9520 | 0.9511 |
| 0.8 | 0.9513 | 0.9490 | 0.9548 | 0.9539 | 0.9516 | 0.9518 |
| 0.9 | 0.9485 | 0.9495 | 0.9586 | 0.9548 | 0.9515 | 0.9615 |

Lower 2.5% confidence limit (nominal coverage 0.975)

| Effectiveness of control regimen(Θ_C_) | AIR (Ψ) | | | | | |
| --- | --- | --- | --- | --- | --- | --- |
|  | 0.5 | 0.6 | 0.7 | 0.8 | 0.9 | 1.0 |
| 0.6 | 0.9749 | 0.9757 | 0.9761 | 0.9755 | 0.9764 | 0.9757 |
| 0.7 | 0.9770 | 0.9762 | 0.9759 | 0.9759 | 0.9755 | 0.9770 |
| 0.8 | 0.9771 | 0.9760 | 0.9763 | 0.9764 | 0.9757 | 0.9770 |
| 0.9 | 0.9759 | 0.9758 | 0.9780 | 0.9758 | 0.9754 | 0.9812 |

Upper 97.5% confidence limit

| Effectiveness of control regimen(Θ_C_) | AIR (Ψ) | | | | | |
| --- | --- | --- | --- | --- | --- | --- |
|  | 0.5 | 0.6 | 0.7 | 0.8 | 0.9 | 1.0 |
| 0.6 | 0.9999 | 0.9977 | 0.9882 | 0.9792 | 0.9768 | 0.9758 |
| 0.7 | 0.9991 | 0.9912 | 0.9779 | 0.9758 | 0.9758 | 0.9770 |
| 0.8 | 0.9945 | 0.9813 | 0.9734 | 0.9755 | 0.9763 | 0.9770 |
| 0.9 | 0.9844 | 0.9782 | 0.9743 | 0.9767 | 0.9792 | 0.9812 |

**5. Cumulative distribution function (CDF) of the lower 5% confidence limit (true AIR=1, 40 expected counterfactual events per arm)**

Delta method, blue line; profile-likelihood method, red line.

Vertical line represents the true AIR (intersection with CDF corresponds to coverage.

Note that the curves are non-smooth because of the discrete sample space.

Control regimen efficacy=0.6


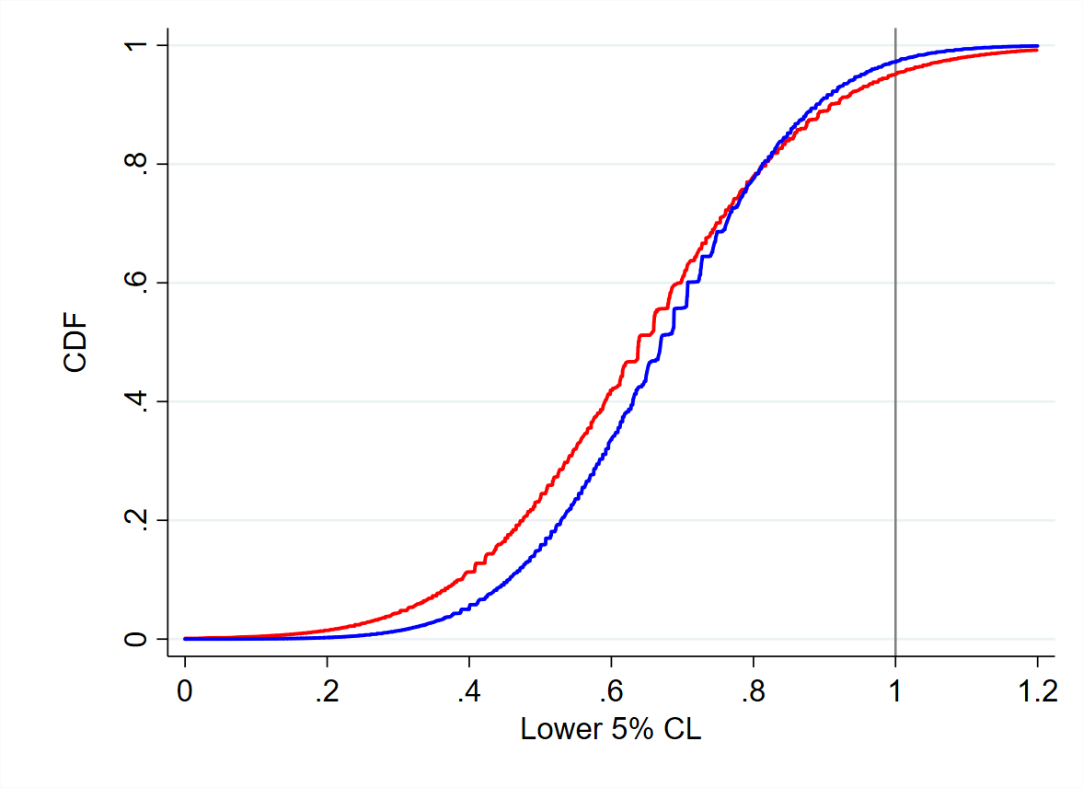


Control regimen efficacy=0.7


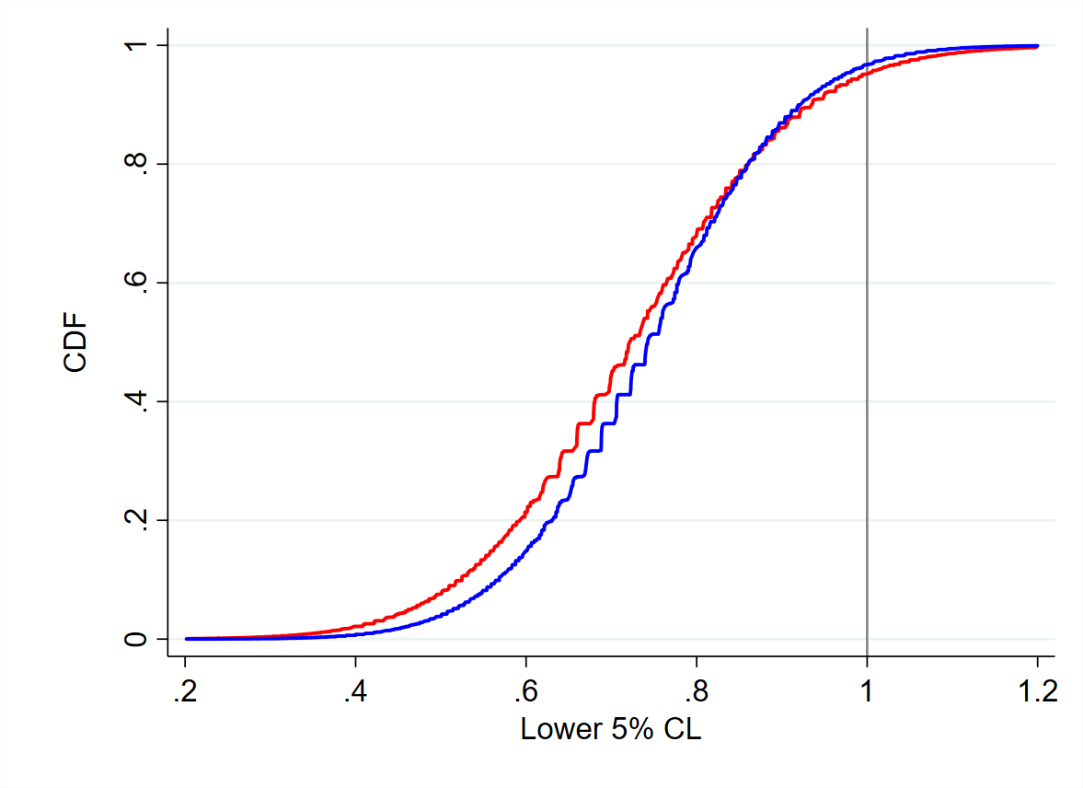


Control regimen efficacy=0.8


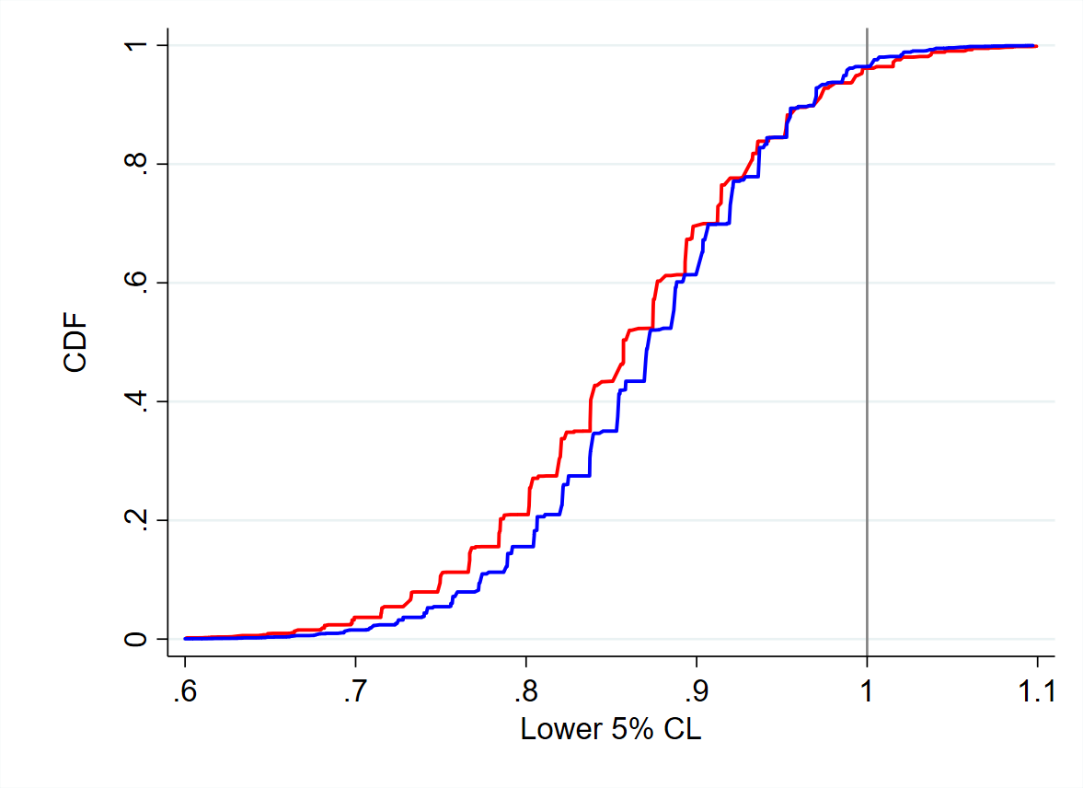


Control regimen efficacy=0.9


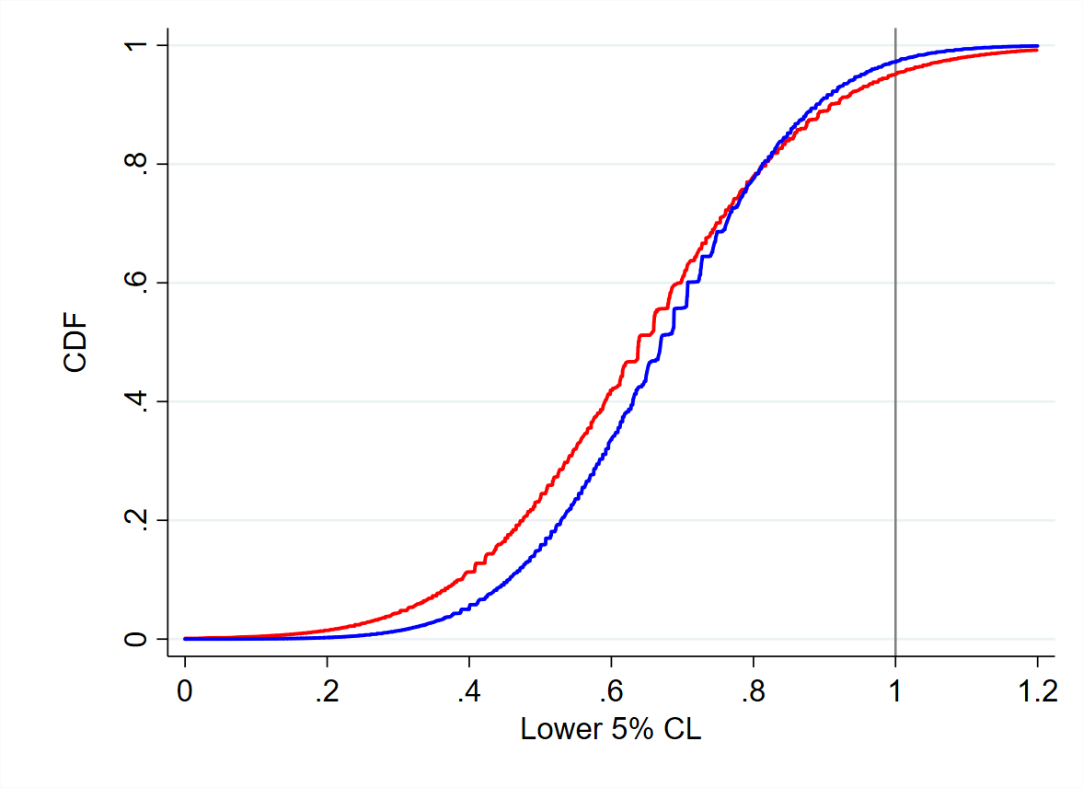

Supplement: Supplementary file 1 — Supplementary Material Details [file j_scid-2021-0002_suppl.docx]
